# Supplementary figures and images for: Metabolic Responses to Manganese Toxicity in Soybean Roots and Leaves
Source: Plants (Basel). 2023 Oct 19;12(20):3615. doi: 10.3390/plants12203615 (PMC10610265; doi:10.3390/plants12203615)

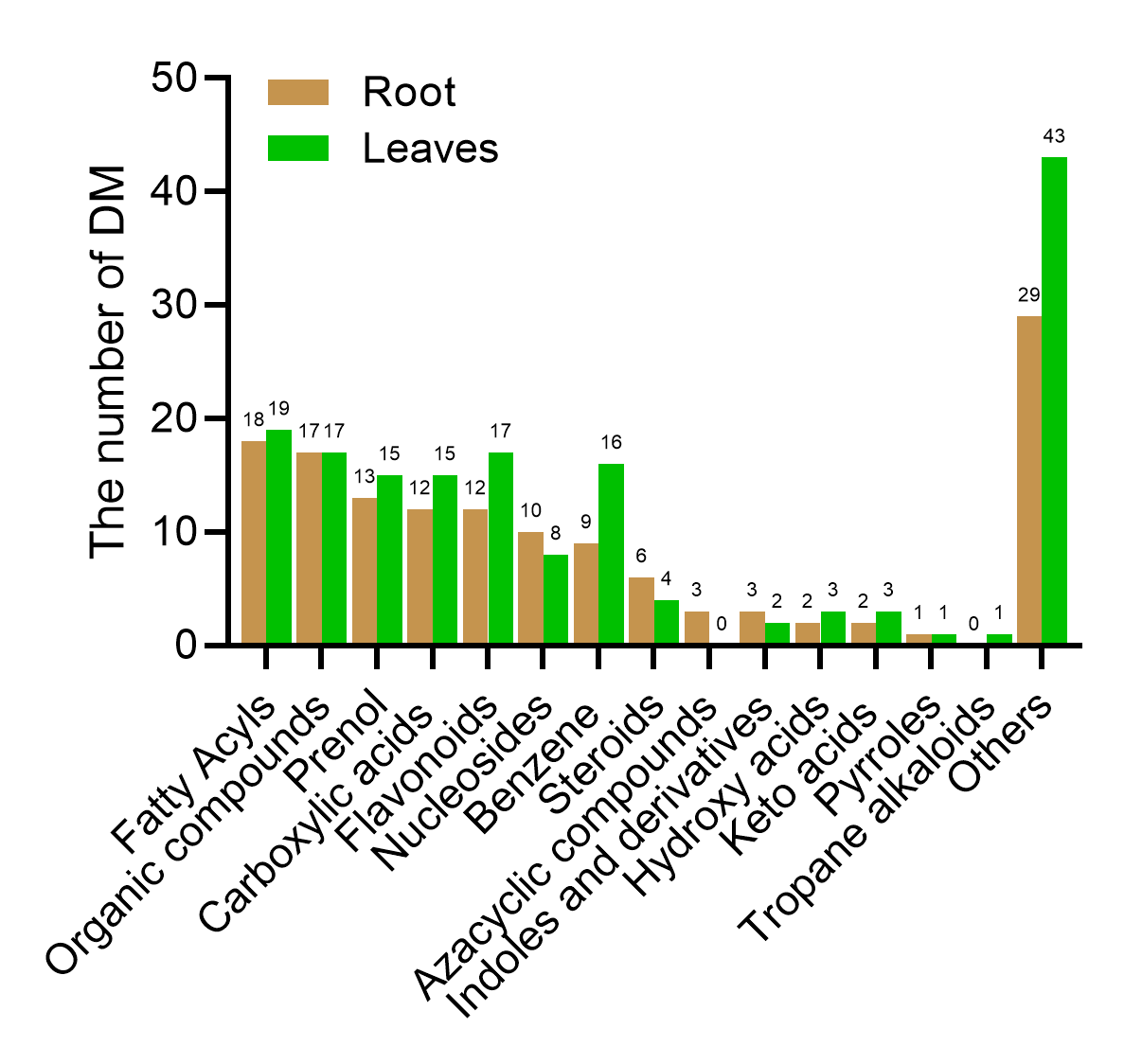

Supplement: Supplementary file 1 [file plants-12-03615-s001.zip › Figure S1.png]

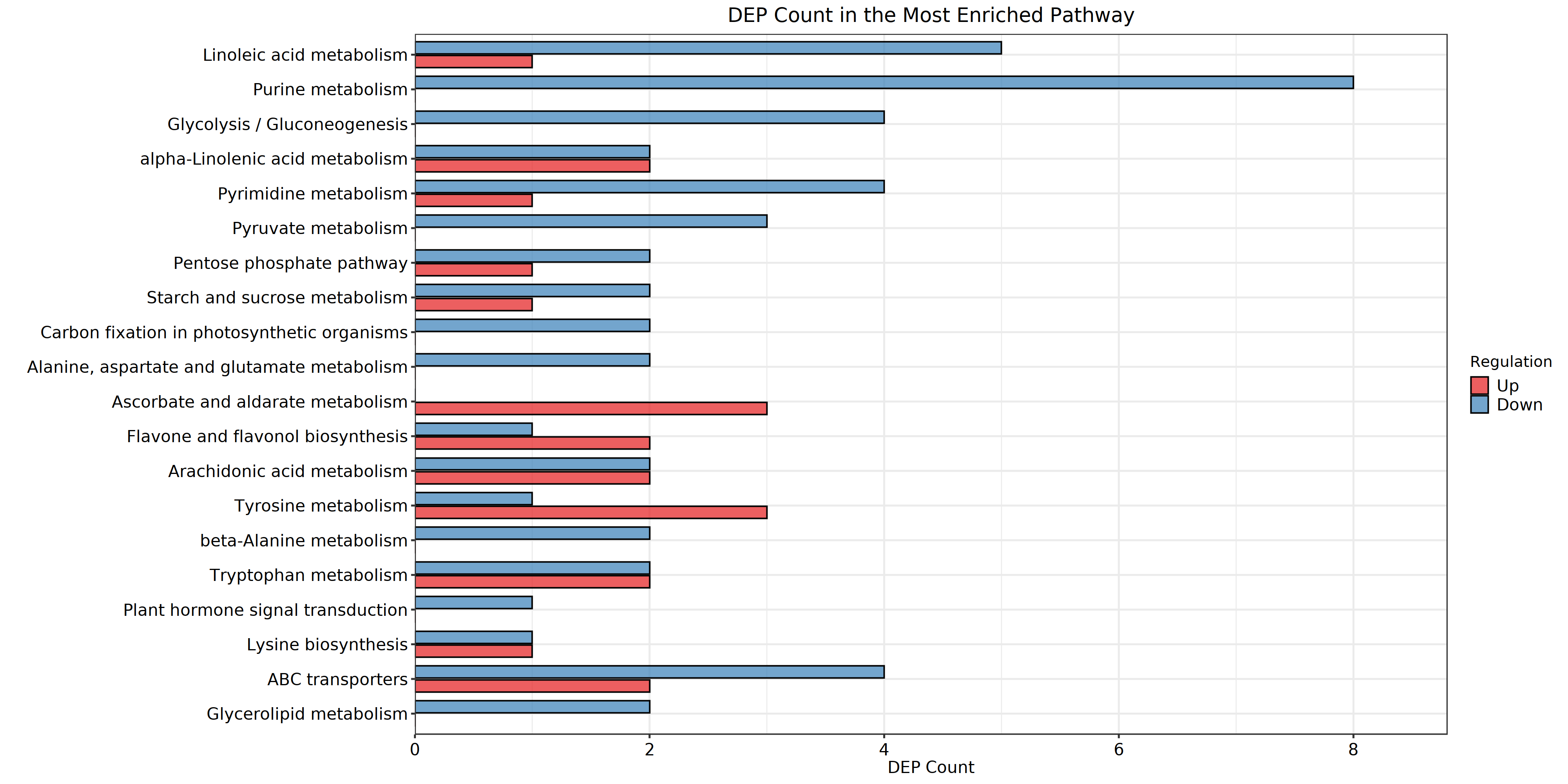

Supplement: Supplementary file 1 [file plants-12-03615-s001.zip › Figure S2.png]

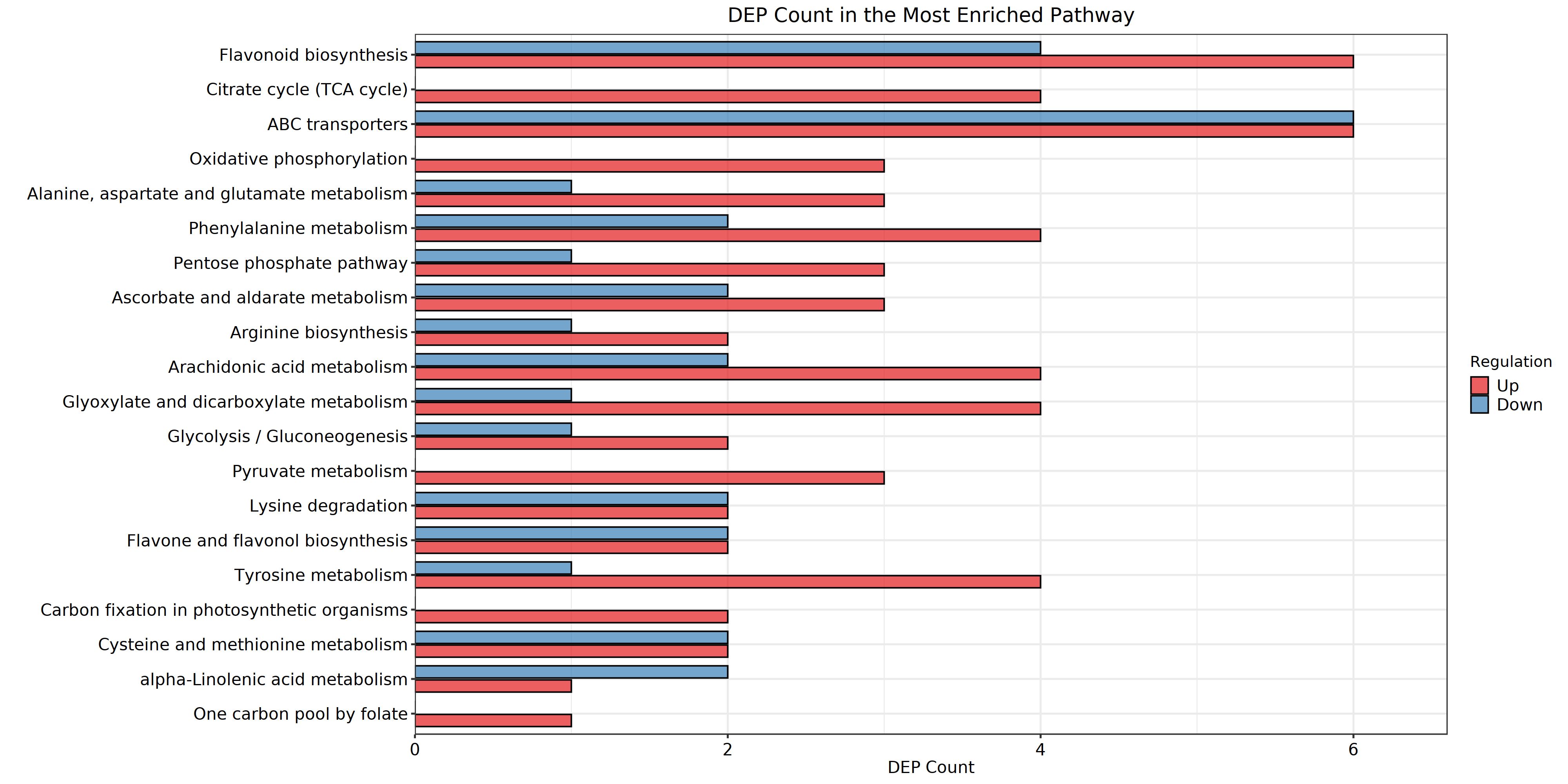

Supplement: Supplementary file 1 [file plants-12-03615-s001.zip › Figure S3.png]

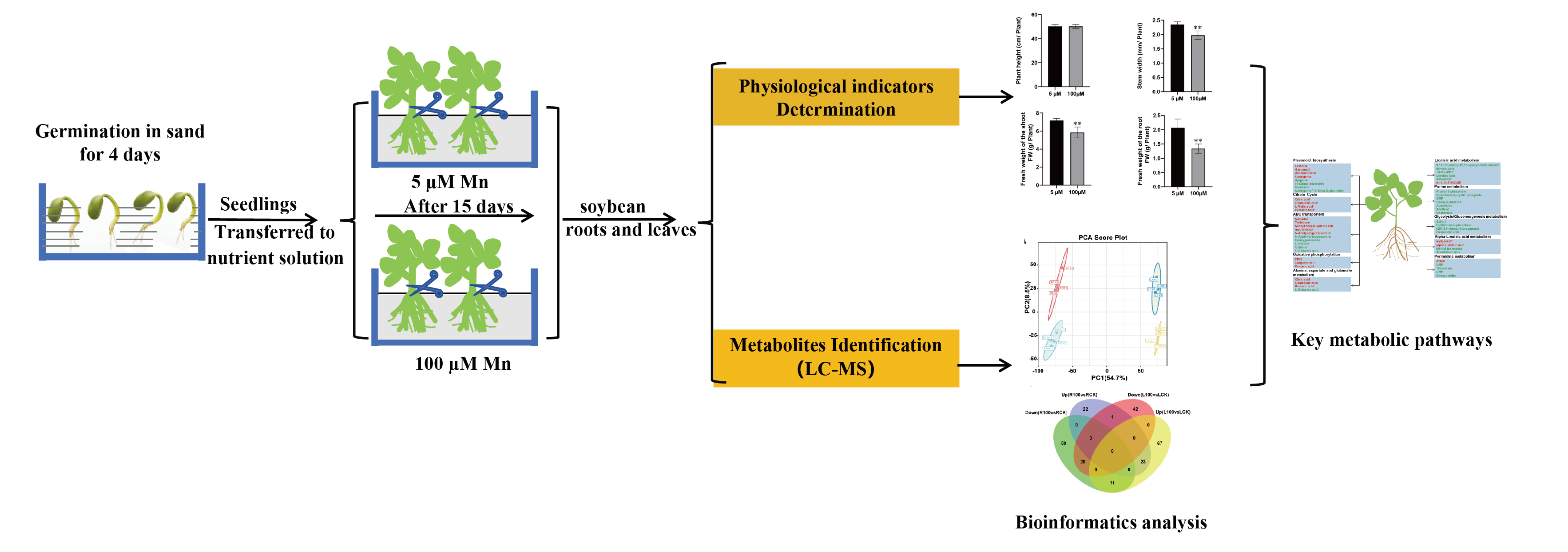

Supplement: Supplementary file 1 [file plants-12-03615-s001.zip › Figure S4.png]
